# Effects of Housing First approaches on health and well-being of adults who are homeless or at risk of homelessness: systematic review and meta-analysis of randomised controlled trials

**DOI:** 10.1136/jech-2018-210981

**Published:** 2019-02-18

**Authors:** Andrew J Baxter, Emily J Tweed, Srinivasa Vittal Katikireddi, Hilary Thomson

**Affiliations:** 1 College of Medicinal, Veterinary and Life Sciences, University of Glasgow, Glasgow, UK; 2 MRC/CSO Social and Public Health Sciences Unit, University of Glasgow, Glasgow, UK

**Keywords:** homelessness, housing first, health, mental illness, substance use

## Abstract

**Background:**

Homelessness is associated with poor health. A policy approach aiming to end homelessness across Europe and North America, the ‘Housing First’ (HF) model, provides rapid housing, not conditional on abstinence from substance use. We aimed to systematically review the evidence from randomised controlled trials for the effects of HF on health and well-being.

**Methods:**

We searched seven databases for randomised controlled trials of interventions providing rapid access to non-abstinence-contingent, permanent housing. We extracted data on the following outcomes: mental health; self-reported health and quality of life; substance use; non-routine use of healthcare services; housing stability. We assessed risk of bias and calculated standardised effect sizes.

**Results:**

We included four studies, all with ‘high’ risk of bias. The impact of HF on most short-term health outcomes was imprecisely estimated, with varying effect directions. No clear difference in substance use was seen. Intervention groups experienced fewer emergency department visits (incidence rate ratio (IRR)=0.63; 95% CI 0.48 to 0.82), fewer hospitalisations (IRR=0.76; 95% CI 0.70 to 0.83) and less time spent hospitalised (standardised mean difference (SMD)=−0.14; 95% CI −0.41 to 0.14) than control groups. In all studies intervention participants spent more days housed (SMD=1.24; 95% CI 0.86 to 1.62) and were more likely to be housed at 18–24 months (risk ratio=2.46; 95% CI 1.58 to 3.84).

**Conclusion:**

HF approaches successfully improve housing stability and may improve some aspects of health. Implementation of HF would likely reduce homelessness and non-routine health service use without an increase in problematic substance use. Impacts on long-term health outcomes require further investigation.

**Trial registration number:**

CRD42017064457

## Background

Access to housing is an important determinant of health, with homeless people having substantially increased morbidity and mortality compared with the housed population.^1 2^ For instance, a recent systematic review found that all-cause mortality in homeless populations in high-income countries is between 3 and 11 times higher than their housed counterparts.^2^ This excess mortality appears to persist even after accounting for socioeconomic deprivation and comorbidity.^3^ Homelessness may have a direct impact on health, through the physical and psychosocial hazards associated with rough sleeping or temporary accommodation (such as excessive cold, heat or damp; physical and sexual violence and other forms of crime); lack of basic amenities and social goods (such as washing facilities); stigma and social isolation and difficulties in accessing healthcare services.^1 4 5^ It is also strongly associated with other experiences deleterious to health, such as poverty (especially child poverty), adverse childhood experiences and substance misuse.^6 7^ The association between homelessness and health is also bidirectional, since poor physical or mental health can increase the risk of unemployment, relationship breakdown and housing loss.^8 9^

Homelessness is increasing across Europe.^10^ Recent increases in homelessness may be linked to economic trends, cuts to public services and welfare benefits and changes in the availability and affordability of housing.^11^ Rehousing homeless (roofless or houseless) persons, or persons at risk of homelessness (insecure housing),^12^ may therefore be an important health intervention.^13–15^ One approach to increasing housing stability is Housing First (HF).

HF is defined in contrast to the traditional ‘Treatment First’ model, which provides temporary accommodation alongside services to address health needs, particularly substance use. The client then progresses to transitional housing before achieving permanent housing; this is conditional on adherence to treatment for mental health and problematic substance use.^16 17^ The ‘HF’ approach aims to assist clients to access permanent housing as an initial step in addressing homelessness. Housing provision is not contingent on compliance with health treatment or substance abstinence. Additionally, HF includes ongoing support, through Intensive Case Management or Assertive Community Treatment approaches.^17 18^ There are a number of established HF projects in North America and Scandinavia, and governments in other countries, including France and the UK, have shown interest in rolling out the model.^19–24^

**Figure 1:**
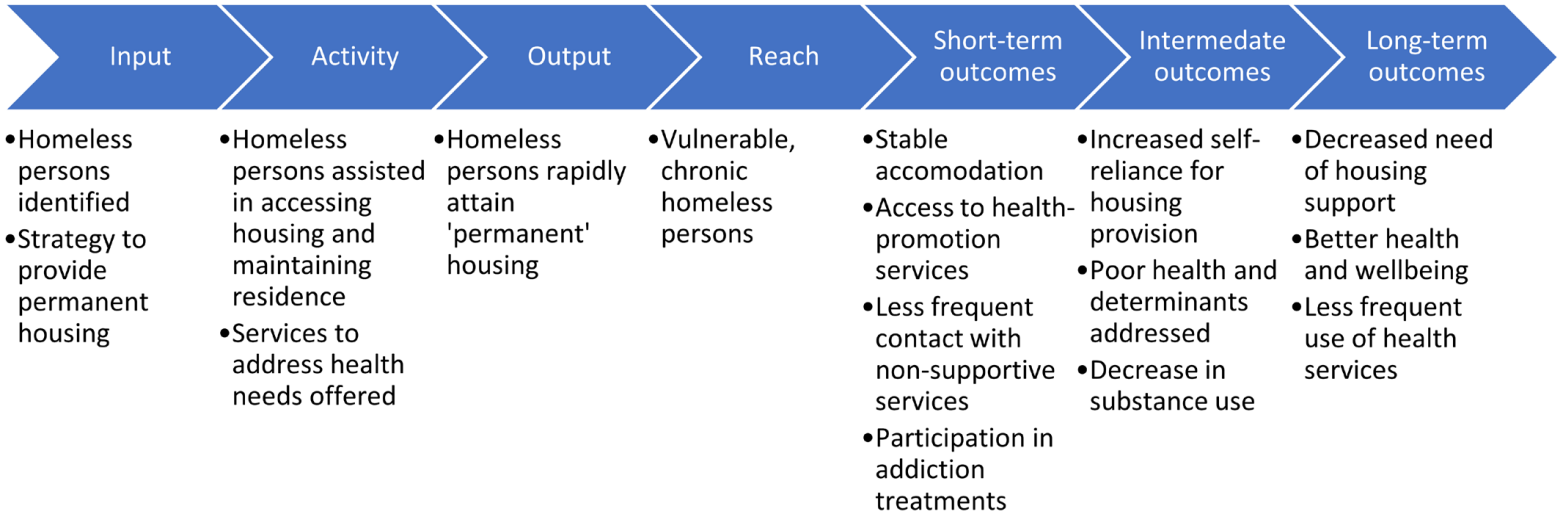
A health-focussed Housing First logic model, adapted from Stergiopoulos *et al* 25 and Tsemberis.^17^

HF may improve health, via the mediating factors of increased housing stability and access to support services (figure 1).^25^ However, critics have suggested that HF may adversely affect health, since engagement with health services is not compulsory and, it is argued, there is therefore a lack of incentive to adhere to treatment or abstain from problematic substance use.

Although prior literature reviews of the impacts of HF have been conducted,^26–29^ these reviews did not meet the reproducibility standards of a systematic review and did not undertake meta-analysis. Moreover, new data on the health impacts of HF are now available. This paper reports the findings of a systematic review of the health effects of the housing provision aspect of HF. The review addresses a current gap in the literature by using a clear definition of the intervention, including recent studies, and conducting the first meta-analyses of health outcome data.

## Methods

We constructed an initial logic model linking HF to health from relevant literature sources (figure 1).^17 25^ We then systematically reviewed evidence of the health effects of HF to test the hypothesis that rapid provision of permanent, non-abstinence-contingent housing to homeless people, leads to health improvement in this vulnerable population compared with housing provision without these features.

The scope, inclusion criteria and methods of the review are outlined below and in box 1. The review protocol was registered on the PROSPERO database.^30^ The intervention was defined in this review as ‘rapid provision of permanent, non-abstinence-contingent housing’. The inclusion of additional supports (such as Intensive Case Management or Assertive Community Treatment) was not used to define the intervention here, as our aim focused on housing. We had intended to compare interventions adhering with the wider principles of HF with interventions providing only housing; however, all studies found included some form of additional support, so this subgroup analysis was not possible. Given all interventions included both rapid provision of permanent, non-contingent housing and additional support, they are therefore labelled ‘HF’, whether or not they were identified as such in the literature.

Box 1 **PICOS criteria used in this review**

*Population*: adults (16 years and older) who meet at least one of the European Typology for Homelessness and Housing Exclusion (ETHOS) criteria: roofless, houseless, living in insecure housing, living in inadequate housing.

*Intervention*: providing the homeless person with access to housing through:

- Assistance in locating and entering housing.
- Subsistence of rental costs to maintain permanent tenancy.

The housing provided was defined as:

- Intended to be permanent—no intention by providers to end or transfer tenancy, counting sustained tenancy as the intended outcome.
- Not contingent on adherence to treatment or substance abstinence.
- Rapid, with the process of securing and entering housing initiated at first contact with the homeless person and with the aim of beginning tenancy promptly.

*Comparators*: treatment as usual groups; although we note that this includes many diverse alternative homeless services and interventions.

*Outcomes*: the primary outcomes, chosen to reflect the aim and research questions, were quantitative measures of health and well-being. These were grouped into five domains:

- Mental health—including self-reported mental health and clinical assessment of mental ill health.
- Self-reported health and quality of life—questionnaires and interviews recording perspectives.
- Substance use—including self-reported occasions of substance use and self-reported problematic substance use.
- Non-routine use of healthcare services—including episodes of hospitalisation and use of emergency services.
- Other, unanticipated measures of health and aspects of well-being associated with health and mental health.

Secondary outcome: housing stability. This included any measure of housing which reflected the stated goals of the intervention of ending homelessness. The use of this domain in the review was based both on the hypothesised causative mechanism leading to changes in health and also its expected availability in almost all studies.

*Study design*: randomised controlled trials

We restricted study types to randomised controlled trials (RCTs), to minimise risk of bias and allow synthesis of data from directly comparable studies. Given a number of RCTs were known to have been conducted, we focused on these as the best available evidence. Primary outcomes were quantitative measures of health, well-being and quality of life; a secondary outcome was housing stability.

### Search strategy

The search strategy was developed in collaboration with a University of Glasgow librarian. The following databases were searched: EMBASE, MEDLINE, PubMed, PsycINFO, Cochrane Central Register of Controlled Trials (CENTRAL), Social Sciences Citation Index and Biosis. Databases were searched using Homeless Persons, Housing and Public Housing as MeSH terms, alongside keywords homeless*, housing and ‘housing first’. Filters were used to select RCTs.^31 32^ The full search strategy for each database is found in online supplementary file 1.

10.1136/jech-2018-210981.supp1 Supplementary data

Searches were restricted to studies published from 1992 (when Pathways to Housing was founded and the intervention first initiated) up to the date of the search (15 May 2017) in peer-reviewed journals. Reference lists of previous reviews were checked for additional studies.

### Screening and selection of studies

Only studies published in English in peer-reviewed journals were included. Only studies which reported a primary health outcome (box 1) were included. Search results were screened by title by one reviewer (AJB) to remove obviously irrelevant citations. Abstracts and full texts were screened independently by two reviewers (AJB and ET). Any discrepancies were resolved by consensus.

### Data extraction and risk of bias assessment synthesis

Data on key study characteristics, intervention details and reported outcome data were extracted by one reviewer (AJB) and checked by a second (ET). Outcome measures from studies were grouped by domain: mental health; quality of life; substance use; non-routine use of healthcare services; housing stability and other health-related outcomes.

To avoid double counting of data, where sampling overlap was stated or suspected for any single outcome or where findings were reported in multiple papers, data were selected to prioritise larger combined samples or allow calculation of standardised effect estimates for comparison with other papers.^33^

The Cochrane Risk of Bias Tool V.2.0,^34^ was used by one researcher (AJB) to assess potential bias for each of the outcomes, and checked by a second (ET). If high risk of bias was reported in at least one domain of bias for an outcome, the outcome was given an overall ‘high’ rating.

### Data synthesis

Calculations of standardised effect sizes were conducted manually in Microsoft Excel.^35^ Standardised mean differences were calculated to compare continuous variables. These were interpreted as ‘small’ to ‘large’ effect size using Cohen’s classification.^36^. Incidence rate ratios were calculated for counts of use of health services, a risk ratio for attaining stable housing and ratios of rate ratios for the substance use subgroup outcome. Where effect sizes were reported only by subgroups and not the whole trial population, data were pooled where possible, otherwise subgroups were presented separately in forest plots.

Forest plots were used to present standardised effect estimates for each outcome domain using Review Manager V.5.3.^37^ A random effects model was used to calculate pooled effect size estimates, 95% CIs and heterogeneity, as we assumed that effect sizes and variation would differ across studies. Where meta-analysis was not possible these were reported narratively in the relevant domain.

Findings were summarised using the Grading of Recommendations Assessment, Development and Evaluation (GRADE) guidance to assess certainty of results for each meta-analysed outcome.^38–42^

## Results

**Figure 2:**
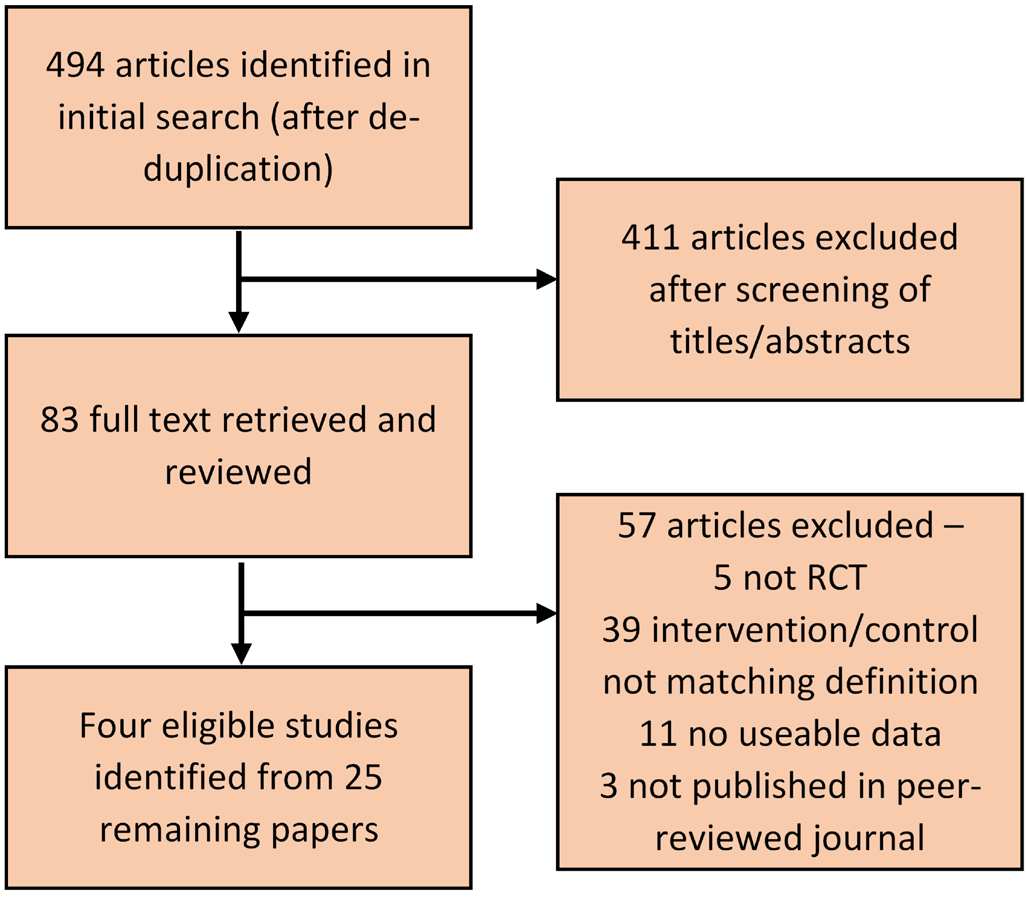
Preferred Reporting Items for Systematic Reviews and Meta-Analyses flow diagram showing literature search and screening process. RCT, randomised controlled trial.

Searching returned 494 records after removal of duplicates (figure 2). Following full-text screening, 25 eligible papers were identified for inclusion; these papers report results from four studies, all based in Canada and the USA (see online supplementary file 2 for included papers and online supplementary file 3 for exclusions).

10.1136/jech-2018-210981.supp2 Supplementary data

10.1136/jech-2018-210981.supp3 Supplementary data

The four studies included in this review are outlined in table 1. The context and ‘treatment as usual’ provision varied across the cities and nations represented in these studies but were not always clearly and fully reported. All participants were homeless or insecurely housed; inadequate housing was not included in the studies retrieved. Beyond the inclusion criteria, there was some variation in the implementation of the HF model. All studies reported a measure of housing stability alongside one or more primary outcome measures. All results are summarised in table 2.

**Table 1:** Overview of included studies

Study	Papers reporting results used in analysis	Duration of study	Location and dates of data collection	Sample size	Participant characteristics	Recruitment methods	Interventions assessed	Treatment as usual used to compare
Pathways Housing First	Gulcur *et al* 8 Tsemberis *et al* 48	24 months	New York City, New York (USA) 1997–2003	225	Homeless, mental health disorder, individual	Recruited from street through referral (157) or psychiatric hospitals (68)	Housing First with ACT	Entered programmes following Treatment First models
At Home/Chez soi	Aubry *et al* 46 Chung *et al* 43 Stergiopoulos *et al* 47	21–24 months	Moncton, Montreal, Toronto, Vancouver, Winnipeg (Canada) 2009–2013	2148	Homeless or insecurely housed, mental health disorder, substance use disorder, individual	Referral from health and social care services, community agencies and institutions	Housing First with ACT; Housing First with ICM; scattered site or congregate	Access to usual services; allowed to use any services other than those offered by Housing First programme
Housing Opportunities for Persons with AIDS	Wolitski *et al* 45	18 months	Baltimore, Maryland; Chicago, Illinois; Los Angeles, California (USA) 2004–2007	630	Homeless or insecurely housed, HIV-positive, individual	Referred by agencies providing HOPWA rental assistance	HOPWA rental assistance with case management	Customary housing services with case management
Chicago Housing for Health Partnership	Sadowski *et al* 44 Buchanan *et al* 52	18 months	Chicago, Illinois (USA) 2003–2007	407	Homeless or insecurely housed, chronic illness, individual	Referral of hospitalised homeless patients by social worker	Short, transitional stay for medical care followed by permanent housing, scattered site or congregate, ongoing case management	Hospital discharge planning service with no continued relationship. Transport to shelter. Access to existing services

ACT, Assertive Community Treatment; HOPWA, Housing Opportunities for Persons With AIDS; ICM, Intensive Case Management.

**Table 2:** GRADE assessment of certainty of estimate of effect size and summary of findings

Outcome		GRADE assessment of certainty*						Summary of findings			Absolute effect estimates		
		Risk of bias	Inconsistency	Indirectness	Imprecision	Publication bias	Certainty	Number of studies	HF/TAU participants	Effect estimate (95% CI)	Outcomes in control†	Outcomes in HF	Effect (95% CI)
Mental health	Self-rated mental health	Serious (−1)	None‡	None	None	Unlikely	+++, moderate	2	1359/1194	SMD=0.07 (−0.19 to 0.33)	14.4—gain in score (out of 100) across 24 months	15.53—gain in score	+1.13 difference in mean change (−3.06 to +5.31)
Quality of life	Self-rated physical health	Serious (−1)	None	None	None	Unlikely	+++, moderate	2	1359/1194	SMD=0.00 (–-0.09 to 0.09)	6.5—gain in score (out of 100) across 24 months	6.5—gain in score (out of 100) across 24 months	no difference in mean change (−1.14 to +1.14)
Substance use	Problematic substance use	Serious (−1)	None	None	None	Unlikely	+++, moderate	2	465/456	*Several outcomes analysed showing no meaningful difference in changes in problematic substance use between groups across 24* months			
Health service use	Hospitalisations	Serious (−1)	None	None	None	Unlikely	+++, moderate	2	516/519	IRR=0.76 (0.70 to 0.83)	1480 hospitalisations per 1000 person-years	1125 hospitalisations per 1000 person-years	355 fewer hospitalisations (252 to 444)
	Time spent hospitalised	Serious (−1)	Serious (−1)	None	None	Unlikely	++, low	3	898/839	SMD=−0.14 (−0.41 to 0.14)	6379 days spent hospitalised per 1000 person-years	6372 days spent hospitalised per 1000 person-years	7 fewer days (7 more days to 20 fewer)
	Emergency department visits	Serious (−1)	None§	None	Serious (−1)¶	Unlikely	++, low**	2	1359/1194	IRR=0.63 (0.48 to 0.82)	2056 emergency department visits per 1000 person-years	1295 emergency department visits per 1000 person-years	761 fewer emergency department visits (370 to 1069)
Housing stability	Attaining stable housing	Serious (−1)	None	None	Serious (−1)¶	Unlikely	++, low	3	985/1000	RR=2.46 (1.58 to 3.84)	37 persons per 100 stably housed at 24 months	75 persons per 100 stably housed at 24 months††	38 more persons stably housed (31 to 46)††
	Time spent stably housed	Serious (−1)	None	None	Serious (−1)¶	Unlikely	++, low	2	1257/1116	SMD=1.24 (0.86 to 1.62)	235 days per person spent stably housed in 24 months	497 days per person spent stably housed in 24 months	261 more days stably housed (181 to 432)

*For all health outcomes, certainty was assessed as either certainty of direction of effect or certainty of null effect. For housing stability outcomes, assessment was made around effect size,^42^

†Control means calculated from all useable control data.

‡High heterogeneity was deemed to be explained by differences in effect by age group, therefore not downgraded.

§All effects in same direction with high confidence of non-null effect; rated ‘none’ despite high I^2^.

¶Score reduction of ‘−1’ here on the basis of relatively small concerns across both inconsistency and imprecision.

**Rated ‘low’ for certainty of effect direction; ‘very low’ for certainty of effect size.

††Absolute effect size recalculated from proportion of each group stably housed.

HF, Housing First; IRR, incidence rate ratio; RR, risk ratio; SMD, standardised mean difference; TAU, treatment as usual.

### Risk of bias

The overall risk of bias was assessed as high for each outcome reported across all four studies (see online supplementary file 4 for all domains and table 2 for overall rating). Bias due to missing outcome data was rated as high if there were no data to evaluate how effectively the effect of loss to follow-up had been addressed.

10.1136/jech-2018-210981.supp4 Supplementary data

### Primary outcomes

#### Mental health

**Figure 3:**
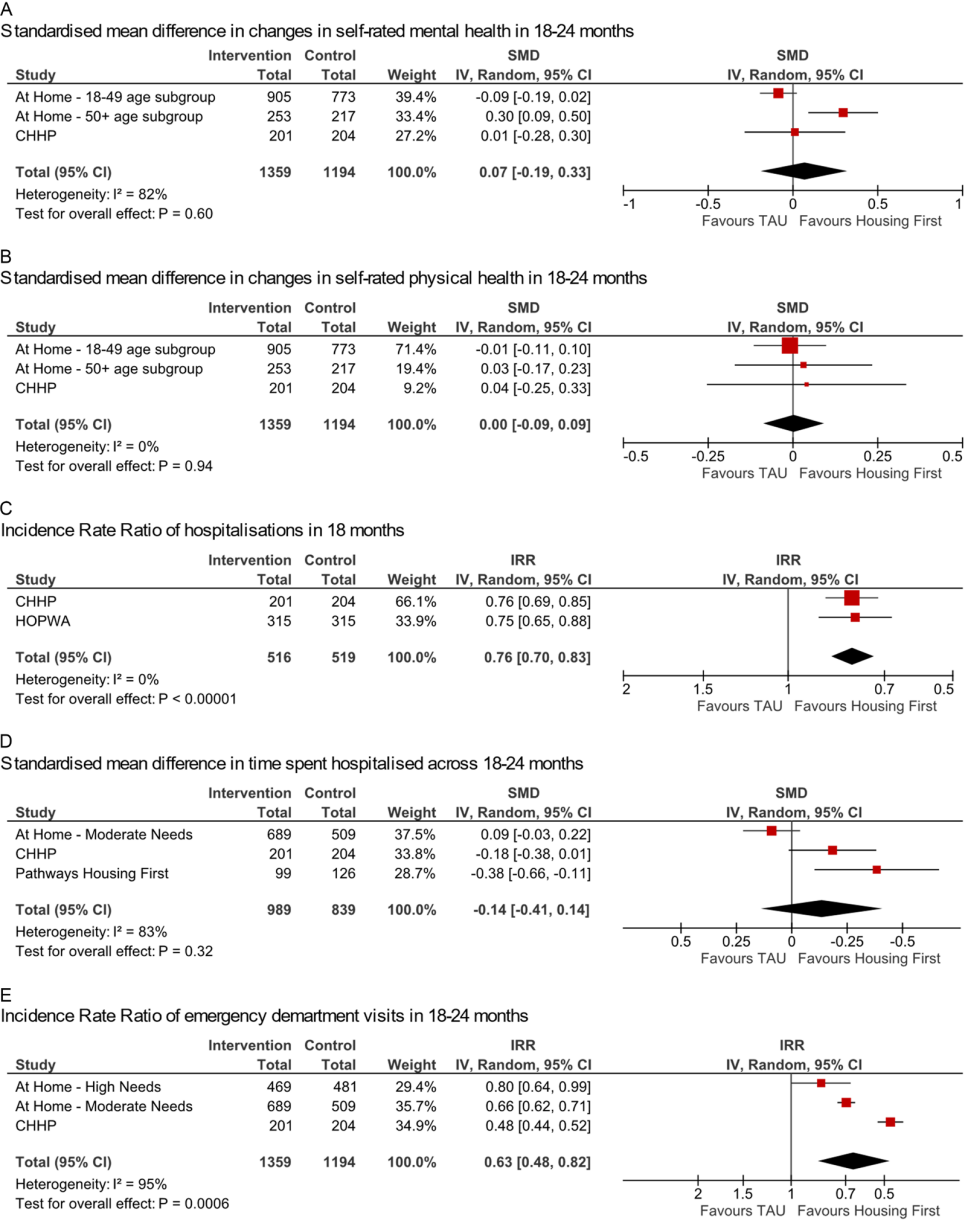
Forest plots presenting standardised effect sizes and meta-analysis of health outcomes comparing intervention and control. At Home—moderate needs results calculated by combining mean counts across four cities reported. CHHP, Chicago Housing for Health Partnership; IRR, incidence rate ratio; SMD, standardised mean difference; TAU, treatment as usual.

All four studies reported mental health outcomes; these were categorised as ‘self-rated mental health’ (n=3: At Home, Chicago Housing for Health Partnership (CHHP) and Housing Opportunities for Persons with AIDS (HOPWA)) and ‘severity of mental health symptoms’ (n=3: At Home, Pathways Housing First (PHF) and HOPWA). Two studies provided data eligible for meta-analysis of self-rated mental health (At Home and CHHP)^43 44^; a very small improvement was seen in intervention groups compared with treatment as usual (SMD=0.07; 95% CI −0.19 to 0.33; p=0.60, I^2^=82%; figure 3A). Additionally, HOPWA reported no statistically significant difference between groups.^45^ Both groups saw improvements in all studies.^43–45^ A small improvement in mental health symptom severity at 24 months in the At Home study was reported (SMD=−0.05; 95% CI −0.31 to 0.22; p=0.73; I^2^=82%).^46 47^ Pathways HF participants saw no significant differences between groups in symptoms over 24 months (F=0.348; p=0.85; no effect direction reported).^48^ Improvements were seen in both intervention and TAU groups of the HOPWA study in depression and perceived stress, with no statistically significant differences between the two conditions.^45^

#### Self-reported health and quality of life

Several measures were reported in the domain of self-reported health and quality of life. Self-rated physical health was reported in three studies (At Home, CHHP and HOPWA).^43–45^ Meta-analysis of two studies showed no detectable difference (SMD=0.00; 95% CI −0.09 to 0.09; p=0.94; I^2^=0%; figure 3B). Participants in both intervention and TAU groups of the HOPWA study reported improvements in self-rated physical health, with no statistically significant difference between groups.^45^ Two measures of quality of life were found in the At Home/Chez Soi study, but not repeated elsewhere. Pooling the two age group subgroups showed a small difference in mean change of generic quality of life between treatment and control groups from baseline, favouring TAU (SMD=−0.03; 95% CI −0.13 to 0.06; p=0.50; I^2^=0%) and a small difference in condition-specific quality of life, favouring intervention (SMD=0.18; 95% CI −0.09 to 0.46; p=0.19; I^2^=83%; not shown).^43^

#### Substance use

Two studies reported substance use outcomes (At Home and PHF).^43 46–49^ Data from PHF were reported as showing no significant differences in either alcohol or drug use at 24 months, but no direction of effect was indicated and so these could not be used in meta-analysis.^48^ Across 48 months, a greater reduction of heavy alcohol use (defined as using alcohol on >28 days in 6 months) in intervention groups compared with control is reported in the study by Padgett *et al*,^49^ with no clear difference in drug use. Pooling the two age group subgroups of the At Home/Chez Soi study showed a very small overall difference in self-reported problematic substance use, favouring HF (ratio of rate ratios=0.96; 95% CI 0.72 to 1.28; p=0.77; I^2^=61%; not shown)^43^; both groups saw decreases in reported problems.^46 47^

#### Health service use

All studies reported a measure of health service use. In meta-analysis (n=2: CHHP and HOPWA), intervention participants experienced fewer hospitalisations (incidence rate ratio (IRR)=0.76; 95% CI 0.70 to 0.83; p<0.00001; I^2^=0%; figure 3C).^44 45^ A small difference was seen in time spent hospitalised, also favouring intervention (n=3: At Home, CHHP and PHF; SMD=−0.14; 95% CI −0.41 to 0.14; p=0.32; I^2^=83%; figure 3D).^8 44 47^

A greater reduction was seen in intervention groups over control groups in number of emergency department visits (n=2: At Home and CHHP; IRR=0.63; 95% CI 0.48 to 0.82; p=0.0006; I^2^=95%; figure 3E).^44 46^ HOPWA participants saw no significant difference between intervention and control groups in likelihood of one or more emergency department visit in each of three 6-month time periods (F=0.63; p=0.5977),^45^ and the CHHP intervention group saw a small reduction in likelihood of one or more emergency department visit in the 18-month period over control (risk ratio (RR)=0.92; 95% CI 0.81 to 1.04).^44^

### Housing stability

All four studies reported measures of housing stability, either recording a proportion of total days reported as ‘stably housed’ or a proportion of the population in stable housing at the end of the trial period. In all four studies, the intervention group was found to have large increases in housing stability over TAU.^43–48^ The combined effects estimate indicated that participants receiving HF are two and a half times more likely to be stably housed after 18–24 months (n=3: At Home, CHHP and HOPWA; RR=2.46; 95% CI 1.58 to 3.84; p<0.00001; I^2^=94%). A large standardised mean difference for time spent housed during trial was also seen, favouring intervention (n=2: At Home and PHF; SMD=1.24; 95% CI 0.86 to 1.62; p<0.0001; I^2^=90%; see online supplementary file 4).

### Subgroups reported

Subgroup comparisons were only conducted in the At Home/Chez Soi study (see online supplementary file 4). In age comparisons, the older group (aged ≥50 years) had better outcomes than the younger group (18–49 years old) in a number of areas, such as self-rated mental health, mental health symptom severity, substance use and quality of life.^43 50^ Participants with less severe mental health and problematic substance use experienced slightly better outcomes.^46 47^ Participants housed together in dedicated accommodation blocks (referred to as the ‘congregate model’) experienced greater improvements than those in ‘scattered site’ housing, in mental health, quality of life and problematic substance use, among other outcomes.^51^ Across all subgroups reported, intervention participants saw large increases in housing stability.

### Other outcomes

Several further outcomes that were related to health were recorded. These are listed in online supplementary file 5. Several small, uncertain effect sizes were observed, favouring HF in most cases, with two of the At Home subgroups experiencing small, uncertain effects favouring treatment as usual.^43 51^ Two studies reported HIV survival and viral load but the findings were conflicting.^45 52^

10.1136/jech-2018-210981.supp5 Supplementary data

## Discussion

### Summary of findings

Our systematic review found that HF resulted in large improvements in housing stability; with unclear short-term impact on health and well-being outcomes. For mental health, quality of life and substance use, no clear differences were seen when compared with TAU. HF participants showed a clear reduction in non-routine use of healthcare services, over TAU. This may be an indicator of improvements in health.

### Comparison with existing literature

The combination of a strong, positive impact on housing with little additional impact on mental health and substance use, compared with TAU, is consistent with the findings of other reviews.^26–29^ Our meta-analyses provide a clear picture of improvements in hospitalisation and emergency department visits, which has not yet been reported in other reviews. Inclusion of only RCTs gives greater confidence that these results are less susceptible to bias. Previous reviews have questioned whether abstinence-contingent housing may lead to greater reductions in problem substance use than HF, although at the cost of housing stability.^28^ However, our results found reductions in problem substance use for both HF and TAU, with no clear difference between them. This is consistent with non-randomised observational evidence suggesting greater effectiveness of HF than TAU in this respect.^16 53^

Prior research on HF suggests that the consumer choice framework allows homeless clients greater perceived control, security and mastery of circumstances, leading to greater improvements in mental health and quality of life.^54 55^ A lack of clear difference seen across the RCTs analysed here may be due to several factors, including the heterogeneity of sample participants, differences in provision of attached services, differences in application of consumer choice and the relatively short-term observation period.

### Strengths and limitations of this review

Our systematic review has several strengths. We conducted a comprehensive search across several databases, which aimed to include all of the relevant studies. The strict use of a clearly predefined protocol, with explicit inclusion and exclusion criteria, has allowed us to bring together all relevant evidence in a transparent manner. This includes drawing on theoretical understanding to define a clearly identifiable and replicable intervention. The use of the logic model allowed testing of the theoretical impact of HF on health through housing stability as a mediator.

This systematic review had some limitations. The scope of this review was primarily limited by the focus on quantitative data from RCTs, and the largest study, a trial of At Home/Chez Soi, carried substantial weight and was the main determinant of effect estimates in rate of emergency department visits, and time spent stably housed. Although trials are underway elsewhere (eg, the Un Chez-Soi d’abord study in France^56 57^), the data included in this review were exclusively from North America and the participants were all selected on the basis of complex health needs (such as mental illness, substance abuse or chronic physical illness) as per the principles of HF.^16 17^ This may limit the generalisability of our findings internationally, as well as to homeless people without complex health needs. Other published data from non-randomised studies are available and may provide further insights into health outcomes, but these studies are at a higher risk of bias. Future qualitative enquiry to identify mechanisms associated with changes in health outcomes could help optimise the benefits of HF.

Across all studies there were high ratings of risk of bias in several areas. Available data were limited to a 24-month follow-up period, providing observations of only short-term outcomes (figure 1). The uncertainty of effect size and direction of the primary health outcomes prevents accurate testing of the hypothesised intermediate and long-term effects of housing stability.

A further systematic review, comparing HF with other interventions, for example, abstinence-contingent housing, housing vouchers, residential treatment and case management (without housing), was published after the completion of this review. This did not consider health outcomes but reported similar results for housing stability.^58^

### Implications for research and implementation

Further questions are prompted by this review which could be addressed by ongoing evaluation of the HF model. Clear reporting of the intervention characteristics (for primary research) and inclusion criteria (for systematic reviews) should be a starting point in future research to ensure testing of an identifiable and replicable model.^59^ Further observations of longer follow-up periods would give greater confidence of impacts on long-term health.

The subgroup analyses of the At Home/Chez Soi study showed several differences in effects for different age groups and health needs. It is unclear if these findings reflect genuine differences^60^; further research would be required to determine if there is greater effectiveness of the intervention for particular groups of homeless persons.

To address some of these concerns, a further systematic review could synthesise the wider evidence base and allow generation of hypotheses about explanations for heterogeneity in reported effects. These data could then be used to refine aspects of HF with the aim of optimising potential beneficial impacts of HF investment. Evaluation of the relative contribution of key principles of HF to its effectiveness would be an important next step. In addition, a clearer differentiation and comparison of the treatments broadly grouped under treatment as usual in this review could show whether better interventions exist for certain groups.

This review adds strength to the calls to adopt HF as an ‘evidence-based’ housing model, having shown consistent improvements in the housing stability of vulnerable homeless persons. Concerns that HF could result in higher rates of problematic substance use than treatment as usual are contradicted by these data. Alongside this, HF could reduce use of non-routine health services, with potential cost savings. Subgroup analysis, although only reported in one study, suggests that housing stability is improved regardless of the age or health needs of the clients, while improvements in health might be differentially seen across groups. According to the logic model in figure 1, the improvements in housing stability associated with HF might be expected to result in intermediate and long-term positive impacts on these and other health outcomes, beyond the timescales considered in this review.

## Conclusion

HF approaches appear to be highly effective in reducing homelessness among vulnerable participants. However, in several direct measurements of short-term health outcomes, the impact of HF is not clear. HF can be seen to reduce non-routine use of healthcare services, which may be an indicator of better health outcomes. Further evidence could be valuable in assessing the long-term effects of improved housing stability on health. HF could be implemented with strong confidence in its success as a housing intervention, alongside some confidence in a lack of immediate adverse effects on health, but with caution in relying on this model for certainty in improved health outcomes.

**What is already known on this subject**

- Homeless people experience very poor health outcomes.
- The Housing First approach aims to provide stable housing to homeless people without imposing requirements on them prior to receiving support.
- Concerns exist that Housing First approaches may result in homeless people maintaining their use of alcohol or other addictive substances.

**What this study adds**

- There is good evidence to support the use of Housing First in providing stable housing to homeless people.
- Housing First does not appear to cause an increase in substance misuse, compared with treatment as usual.
- Housing First approaches do not appear to consistently improve or harm health in the short-term, but long-term impacts are unknown.
