# Supplementary material for: Effects of Housing First approaches on health and well-being of adults who are homeless or at risk of homelessness: systematic review and meta-analysis of randomised controlled trials
Source: J Epidemiol Community Health. 2019 Feb 18;73(5):379–87. doi: 10.1136/jech-2018-210981 (PMC6581117; doi:10.1136/jech-2018-210981)
Supplement: Supplementary data [file jech-2018-210981supp001.pdf]

Supplementary File 1 – Search strategy

- Population
  - MeSH – Homeless Persons (exploded)
  - Keywords – homeless*, insecure* hous*, unstabl* hous*, inadequate* hous*, precarious* hous*, houseless, roofless
- Intervention
  - MeSH – Housing, Public Housing (exploded)
  - Keywords – housing, 'housing first'
- Control – N/A
- Outcome – N/A – looking at multiple outcomes
- Study design – Randomised controlled trials (Cochrane/SIGN filters)

Search from 1992

**Database**	**Search terms**
Medline (Ovid)	1. exp Homeless Persons/
	2. homeless*.mp.
	3. (inadequate* adj2 hous*).mp.
	4. (insecure* adj2 hous*).mp.
	5. (precarious* adj2 hous*).mp.
	6. (unstabl* adj2 hous*).mp.
	7. houseless*.mp.
	8. roofless*.mp.
	9. 1 or 2 or 3 or 4 or 5 or 6 or 7 or 8
	10. exp Housing/
	11. exp Public Housing/
	12. housing.mp.
	13. "housing first".mp.
	14. 10 or 11 or 12 or 13
	15. 'randomized controlled trial'.pt.
	16. randomized.ab.
	17. randomly.ab.
	18. trial.ab.
	19. Randomized Controlled Trials as Topic/
	20. randomized controlled trial/
	21. random allocation/
	22. randomly allocated.tw.
	23. (allocated adj2 random$).tw.
	24. 15 or 16 or 17 or 18 or 19 or 20 or 21 or 22 or 23
	25. 9 and 14 and 24

Embase (Ovid)	1. exp Homeless Persons/<br>2. homeless*.mp.<br>3. (inadequate* adj2 hous*).mp.<br>4. (insecure* adj2 hous*).mp.<br>5. (precarious* adj2 hous*).mp.<br>6. (unstabl* adj2 hous*).mp.<br>7. houseless*.mp.<br>8. roofless*.mp.<br>9. 1 or 2 or 3 or 4 or 5 or 6 or 7 or 8<br>10. exp Housing/<br>11. exp Public Housing/<br>12. housing.mp.<br>13. “housing first”.mp.<br>14. 10 or 11 or 12 or 13<br>15. Randomized controlled trial/<br>16. exp Randomization/<br>17. 'randomi?ed controlled trial$'.tw.<br>18. rct.tw.<br>19. (random$ adj2 allocat$).tw.<br>20. 15 or 16 or 17 or 18 or 19<br>21. 9 and 14 and 20
PubMed	((homeless persons[MeSH Terms]) OR homeless* OR ((inadequate* OR insecure* OR precarious* OR unstabl*) AND (housed OR housing)) OR houseless* OR roofless*)<br>AND<br>(housing[MeSH Terms] OR public housing[MeSH Terms] OR housing[tiab] OR ("housing first"))<br>AND<br>((randomized controlled trial[pt]) OR randomized[tiab] OR randomly[tiab] OR trial[tiab])
Social Sciences Citation Index (Web of Science)<br>Biosis Citation Index (Web of Science)	TS=(homeless* or ‘inadequate* hous*’ 'insecure* hous*' or 'precarious* hous*' or 'unstabl* hous*' or houseless* or roofless*)<br>and<br>TS=(housing or 'housing first’)<br>and<br>TS=('randomized controlled trial$' or ('randomized controlled' NEAR trial*) or (random* NEXT/2 allocat*))
PsycINFO	S1. TX homeless*<br>S2. TX 'inadequate* hous*'<br>S3. TX 'insecure* hous*'<br>S4. TX ‘precarious* hous*’<br>S5. TX 'unstabl* hous*'<br>S6. TX houseless*<br>S7. TX roofless*<br>S8. S1 OR S2 OR S3 OR S4 OR S5 OR S6 OR S7<br>S9. MA Housing OR DE "Housing" OR DE "Assisted Living" OR DE "Dormitories" OR DE "Group Homes" OR DE "Shelters"<br>S10. TX housing*<br>S11. TX ‘housing first’

	S12.	S9 OR S10 OR S11
	S13.	TX randomi* control* trial*
	S14.	TX random*
	S15.	S13 OR S14
	S16.	S8 AND S12 AND S15
Cochrane Central Register of Controlled Trials	#1	MeSH descriptor: [Homeless Persons] explode all trees
	#2	homeless*
	#3	inadequate* hous*
	#4	insecure* hous*
	#5	precarious* hous*
	#6	unstabl* hous*
	#7	housless*
	#8	roofless*
	#9	#1 or #2 or #3 or #4 or #5 or #6 or #7 or #8
	#10	MeSH descriptor: [Housing] explode all trees
	#11	MeSH descriptor: [Public Housing] explode all trees
	#11	housing*
	#12	"housing first"
	#14	#10 or #11 or #12 or #13
	#15	randomised controlled trial
	#16	MeSH descriptor: [Randomized Controlled Trial] explode all trees
	#17	#15 or #16
	#18	#9 and #14 and #17
