# Supplementary material for: Effects of Housing First approaches on health and well-being of adults who are homeless or at risk of homelessness: systematic review and meta-analysis of randomised controlled trials
Source: J Epidemiol Community Health. 2019 Feb 18;73(5):379–87. doi: 10.1136/jech-2018-210981 (PMC6581117; doi:10.1136/jech-2018-210981)
Supplement: Supplementary data [file jech-2018-210981supp002.pdf]

Supplementary File 2 – Included studies

Study: **Pathways Housing First** (PHF)

Location	Study design	Data collection	Outcome domains	Participant characteristics	Interventions assessed
New York City, NY (USA)	Randomised controlled trial	1997 to 2003	Housing Stability, Substance Use, Mental Health, Health Service Use	Homeless, mental health disorder, individual	Housing First with ACT

Papers reporting this study

Authors and year	No. participants	Subgroups reported	Outcome domains synthesised	Data reported time points
Papers included in meta-analysis				
Gulcur et al. 2003[1]	225	Recruited from street/hospital	Health Service Use	24 months
Tsemberis et al. 2004[2]	225		Housing Stability, Substance Use, Mental Health	24 months
Other papers				
Padgett et al. 2006[3]	225			Baseline to 48 months

Study: **At Home/Chez Soi**

Location	Study design	Data collection	Outcome domains	Participant characteristics	Interventions assessed
Moncton, Montreal, Toronto, Vancouver, Winnipeg (Canada)	Randomised Controlled Trial	2009 to 2013	Housing Stability, Substance Use, Mental Health, Health Service Use, Quality of Life	Homeless, mental health disorder, substance use disorder, individual	Housing First with ACT, Housing First with ICM, Congregate Housing First

Papers reporting this study

Authors and year	No. participants	Subgroups reported	Outcome domains synthesised	Data reported time points
Papers included in meta-analysis				
Aubry et al. 2016[4]	950		Housing Stability, Substance Use, Mental Health, Quality of Life	6 months, 12 months, 18 months, 24 months
Chung et al. 2017[5]	2148	Aged ≥50, aged 18-49	Housing Stability, Substance Use, Mental Health, Quality of Life	12 months, 24 months
Stergiopoulos, Hwang, et al. 2015[6]	1198	City A, B, C, D	Health Service Use, Housing Stability, Substance Use, Mental Health, Quality of Life	6 months, 12 months, 18 months, 24 months

Other papers				
Adair et al. 2017[7]	2140			24 months
Aubry et al. 2015[8]	950		Mental Health	6 months, 12 months
Kirst et al. 2015[9]	575			6 months, 12 months, 18 months, 24 months
Kozloff, Adair, et al. 2016[10]	156		Housing Stability, Substance Use, Mental Health, Quality of Life, Health Service Use	6 months, 12 months, 18 months, 24 months
O'Campo et al. 2016[11]	197			6 months, 12 months, 18 months, 24 months
Palepu, Patterson, Moniruzzaman, Frankish, et al. 2013[12]	497	Presence/absence substance dependence		12 months
Parpouchi et al. 2016[13]	497		Other (sexual behaviour)	6 months, 12 months, 18 months, 24 months
Patterson, Moniruzzaman, et al. 2013[14]	497	Congregate for high-needs, HF+ACT for high-		6 months, 12 months

		needs, HF+ICM for moderate needs		
Patterson et al. 2014[15]	497	Congregate for high-needs, HF+ACT for high-needs, HF+ICM for moderate needs	Other (Community and psychological integration)	6 months, 12 months
Powell et al. 2017[16]	1186			6 months, 12 months, 18 months, 24 months
Rezansoff et al. 2016[17]	165	Congregate, Scattered Site	Other (medication adherence for psychosis)	24 months
Somers et al. 2015[18]	497	Congregate for high-needs, HF+ACT for high-needs, HF+ICM for moderate needs; Substance use at baseline or not		6 months, 12 months, 18 months, 24 months
Somers et al. 2017[19]	297	Congregate, Scattered Site	Housing Stability, Substance Use, Mental Health, Quality of Life	24 months
Stergiopoulos, Gozdzik, et al. 2015[20]	378			24 months
Stergiopoulos et al. 2016[21]	237			12 months, 24 months

Woodhall-Melnik et al. 2015[22]	575	High needs, moderate needs	Other (BMI, Waist circumference)	24 months

Study: **Housing Opportunities for Persons With AIDS** (HOPWA)

Location	Study design	Data collection	Outcome domains	Participant characteristics	Interventions assessed
Baltimore, MD; Chicago, IL; Los Angeles, CA (USA)	Randomised Controlled Trial	2004 to 2007	Housing Stability, Mental Health, Health Service Use	Homeless, HIV-positive, individual	Non-contingent housing, scattered site format, time-unlimited rent subsidy

Papers reporting this study

Authors and year	No. participants	Subgroups reported	Outcome domains synthesised	Data reported time points
Papers included in meta-analysis				
Wolitski et al. 2010[23]	630	None	Housing Stability, Mental Health, Health Service Use, Other (Health risk behaviour)	6 months, 12 months, 18 months

Study: **Chicago Housing for Health Partnership** (CHHP)

Location	Study design	Data collection	Outcome domains	Participant characteristics	Interventions assessed
Chicago, Il (USA)	Randomised Controlled Trial	2003 to 2007	Mental Health, Quality of Life, Health Service Use	Homeless, chronic illness (inc. HIV-positive), individual	Non-contingent housing, scattered site or congregate, time-unlimited rent subsidy

Papers reporting this study

Authors and year	No. participants	Subgroups reported	Outcome domains synthesised	Data reported time points
Papers included in meta-analysis				
Sadowski et al. 2009[24]	407	None	Mental Health, Quality of Life, Health Service Use	18 months
Other papers				
Buchanan et al. 2009[25]	105	None		12 months

References

1. Gulcur L, Stefancic A, Shinn M, et al. Housing, hospitalization and cost outcomes for homeless individuals with psychiatric disabilities participating in continuum of care and housing first programmes. *J Community Appl Soc Psychol* 2003;13(2):171-86 doi:10.1002/casp.723 [published Online First: 9 April 2003].
2. Tsemberis S, Gulcur L, Nakae M. Housing First, Consumer Choice, and Harm Reduction for Homeless Individuals With a Dual Diagnosis. *Am J Public Health* 2004;94(4):651-56 doi:10.2105/AJPH.94.4.651 [published Online First: 10 October 2010].

3. Padgett DK, Gulcur L, Tsemberis S. Housing first services for people who are homeless with co-occurring serious mental illness and substance abuse. *Research on Social Work Practice* 2006;16(1):74-83 doi:10.1177/1049731505282593.
4. Aubry T, Goering P, Veldhuizen S, et al. A multiple-city RCT of housing first with assertive community treatment for homeless Canadians with serious mental illness. *Psychiatr Serv* 2016;67:275-81 doi:10.1176/appi.ps.201400587 [published Online First: 1 December 2015].
5. Chung TE, Gozdzik A, Palma Lazgare LI, et al. Housing first for older homeless adults with mental illness: A subgroup analysis of the at home/chez soi randomized controlled trial. *Int J Geriatr Psychiatry* 2017 doi:10.1002/gps.4682 [published Online First: 16 February 2017].
6. Stergiopoulos V, Hwang SW, Gozdzik A, et al. Effect of Scattered-Site Housing Using Rent Supplements and Intensive Case Management on Housing Stability Among Homeless Adults With Mental Illness A Randomized Trial. *Jama-Journal of the American Medical Association* 2015;313(9):905-15 doi:10.1001/jama.2015.1163.
7. Adair CE, Streiner DL, Barnhart R, et al. Outcome trajectories among homeless individuals with mental disorders in a multisite randomised controlled trial of housing first. *The Canadian Journal of Psychiatry / La Revue canadienne de psychiatrie* 2017;62:30-39 doi:10.1177/0706743716645302.
8. Aubry T, Tsemberis S, Adair CE, et al. One-Year Outcomes of a Randomized Controlled Trial of Housing First With ACT in Five Canadian Cities. *Psychiatr Serv* 2015;66(5):463-69 doi:10.1176/appi.ps.201400167.
9. Kirst M, Zerger S, Misir V, et al. The impact of a Housing First randomized controlled trial on substance use problems among homeless individuals with mental illness. *Drug Alcohol Depend* 2015;146:24-29 doi:10.1016/j.drugalcdep.2014.10.019.
10. Kozloff N, Adair CE, Lazgare LIP, et al. "Housing First" for Homeless Youth With Mental Illness. *Pediatrics* 2016;138:e20161514 doi:10.1542/peds.2016-1514 [published Online First: 30 September 2016].
11. O'Campo P, Stergiopoulos V, Nir P, et al. How did a Housing First intervention improve health and social outcomes among homeless adults with mental illness in Toronto? Two-year outcomes from a randomised trial. *BMJ Open* 2016;6(9):e010581 doi:10.1136/bmjopen-2015-010581 [published Online First: 2016/09/14].
12. Palepu A, Patterson ML, Moniruzzaman A, et al. Housing First Improves Residential Stability in Homeless Adults With Concurrent Substance Dependence and Mental Disorders. *Am J Public Health* 2013;103(Suppl. 2):E30-E36 doi:10.2105/ajph.2013.301628.
13. Parpouchi M, Moniruzzaman A, McCandless L, et al. Housing First and Unprotected Sex: A Structural Intervention. *J Health Care Poor Underserved* 2016;27(3):1278-302 doi:10.1353/hpu.2016.0113 [published Online First: 2016/08/16].
14. Patterson ML, Moniruzzaman A, Palepu A, et al. Housing First improves subjective quality of life among homeless adults with mental illness: 12-month findings from a randomized controlled trial in Vancouver, British Columbia. *Soc Psychiatry Psychiatr Epidemiol* 2013;48:1245-59 doi:10.1007/s00127-013-0719-6.
15. Patterson ML, Moniruzzaman A, Somers JM. Community participation and belonging among formerly homeless adults with mental illness after 12 months of Housing First in Vancouver, British Columbia: A randomized controlled trial. *Community Ment Health J* 2014;50(5):604-11 doi:10.1007/s10597-013-9672-9.
16. Powell GA, Adair CE, Streiner DL, et al. Changes in quality of life from a homelessness intervention: true change, response shift, or random variation. *Qual Life Res* 2017;26:1853-64 doi:10.1007/s11136-017-1522-8.

17. Rezansoff SN, Moniruzzaman A, Fazel S, et al. Housing First Improves Adherence to Antipsychotic Medication Among Formerly Homeless Adults With Schizophrenia: Results of a Randomized Controlled Trial. *Schizophr Bull* 2016 doi:10.1093/schbul/sbw136 [published Online First: 2016/09/25].
18. Somers JM, Moniruzzaman A, Palepu A. Changes in daily substance use among people experiencing homelessness and mental illness: 24-month outcomes following randomization to Housing First or usual care. *Addiction* 2015;110(10):1605-14 doi:10.1111/add.13011.
19. Somers JM, Moniruzzaman A, Patterson M, et al. A Randomized Trial Examining Housing First in Congregate and Scattered Site Formats. *PLoS One* 2017;12(1):e0168745 doi:10.1371/journal.pone.0168745 [published Online First: 11 Jan 2017].
20. Stergiopoulos V, Gozdzik A, Misir V, et al. Effectiveness of Housing First with Intensive Case Management in an Ethnically Diverse Sample of Homeless Adults with Mental Illness: A Randomized Controlled Trial. *PLoS One* 2015;10(7):e0130281 doi:10.1371/journal.pone.0130281.
21. Stergiopoulos V, Gozdzik A, Misir V, et al. The effectiveness of a Housing First adaptation for ethnic minority groups: findings of a pragmatic randomized controlled trial. *BMC Public Health* 2016;16:1110 doi:10.1186/s12889-016-3768-4.
22. Woodhall-Melnik J, Misir V, Kaufman-Shriqui V, et al. The Impact of a 24 Month Housing First Intervention on Participants' Body Mass Index and Waist Circumference: Results from the At Home/Chez Soi Toronto Site Randomized Controlled Trial. *PLoS One* 2015;10(9):e0137069 doi:10.1371/journal.pone.0137069.
23. Wolitski RJ, Kidder DP, Pals SL, et al. Randomized trial of the effects of housing assistance on the health and risk behaviors of homeless and unstably housed people living with HIV. *AIDS Behav* 2010;14(3):493-503 doi:10.1007/s10461-009-9643-x [published Online First: 1 December 2009].
24. Sadowski LS, Kee RA, VanderWeele TJ, et al. Effect of a housing and case management program on emergency department visits and hospitalizations among chronically ill homeless adults: A randomized trial. *JAMA: Journal of the American Medical Association* 2009;301(17):1771-78 doi:10.1001/jama.2009.561.
25. Buchanan D, Kee R, Sadowski LS, et al. The health impact of supportive housing for HIV-positive homeless patients: a randomized controlled trial. *Am J Public Health* 2009;99 Suppl 3:S675-80 doi:10.2105/AJPH.2008.137810 [published Online First: 16 April 2009].
