# Supplementary material for: Effects of Housing First approaches on health and well-being of adults who are homeless or at risk of homelessness: systematic review and meta-analysis of randomised controlled trials
Source: J Epidemiol Community Health. 2019 Feb 18;73(5):379–87. doi: 10.1136/jech-2018-210981 (PMC6581117; doi:10.1136/jech-2018-210981)
Supplement: Supplementary data [file jech-2018-210981supp003.pdf]

Supplementary File 3 – Studies excluded at full text screening

Paper	Reason for exclusion
Adair et al. 2016[1]	Randomisation conducted in sampling of positive outcomes after intervention
Aubry et al. 2015[2]	No relevant outcomes reported
Burnam et al. 1995[3]	Intervention not qualifying – residence provision was not non-contingent or permanent
Caplan et al. 2006[4]	Control not qualifying
Cheng et al. 2007[5]	Intervention not qualifying – housing voucher can be denied in cases of continued use of controlled substances (section 8 housing voucher) [58]
Conrad et al. 1998[6]	Intervention not qualifying – residence not permanent
Dickey et al. 1996[7]	Control not qualifying
Dickey et al. 1997[8]	Control not qualifying
Edens et al. 2011[9]	Not a Randomised Controlled Trial
Fletcher et al. 2008[10]	Intervention not qualifying – housing provision not defined as core element
Forchuk et al. 2008[11]	Intervention not qualifying – Contingent on already having achieved sobriety
Fowler & Chavira 2014[12]	Intervention not qualifying – housing voucher can be denied in cases of continued use of controlled substances (section 8 housing voucher) [58]
Fowler & Schoeny 2015[13]	Intervention not qualifying – housing voucher can be denied in cases of continued use of controlled substances (section 8 housing voucher) [58]
Geller 2014[14]	Not a Randomised Controlled Trial
Gewirtz et al. 2015[15]	Control not qualifying
Goldfinger et al. 1999[16]	Control not qualifying
Greenwood et al. 2005[17]	No relevant outcomes reported
Gulcur et al. 2007[18]	No relevant outcomes reported
Guo et al. 2016[19]	Control not qualifying
Hurlburt et al. 1996[20]	Intervention not qualifying – housing voucher can be denied in cases of continued use of controlled substances (section 8 housing voucher) [58]
Kertesz et al. 2007[21]	Intervention not qualifying – not providing permanent housing
Korr & Joseph 1995[22]	Intervention not qualifying – not providing permanent housing
Kozloff 2016[23]	Not published in a peer-reviewed journal – conference paper
Kozloff, Stergiopoulos, Adair, et al. 2016[24]	Not published in a peer-reviewed journal – conference paper
Krabbenborg et al. 2015[25]	Intervention not qualifying

Lapham et al. 1993[26]	Intervention not qualifying – not providing permanent housing
Lapham et al. 1995[27]	Intervention not qualifying – not providing permanent housing
Levitt et al. 2013[28]	Intervention not qualifying – housing provision not ‘rapid’ (prior transitionary stage in shelter required)
Malte et al. 2017[29]	Intervention not qualifying – not providing permanent housing
Mares & Rosenheck 2011[30]	Not a Randomised Controlled Trial
McHugo et al. 2004[31]	Control not qualifying
Milby et al. 2005[32]	Intervention not qualifying – not providing permanent housing
Morse et al. 1994[33]	Intervention not qualifying – not providing permanent housing
Morse et al. 2006[34]	Intervention not qualifying – not providing permanent housing
Morse et al. 2008[35]	Intervention not qualifying – not providing permanent housing
Nelson et al. 2015[36]	No relevant outcomes reported
O’Connell et al. 2008[37]	Intervention not qualifying – housing voucher can be denied in cases of continued use of controlled substances (section 8 housing voucher) [58]
O’Connell et al. 2012[38]	Intervention not qualifying – housing voucher can be denied in cases of continued use of controlled substances (section 8 housing voucher) [58]
O’Connell et al. 2017[39]	Intervention not qualifying – housing voucher can be denied in cases of continued use of controlled substances (section 8 housing voucher) [58]
Palepu et al. 2013[40]	Intervention not qualifying – housing voucher can be denied in cases of continued use of controlled substances (section 8 housing voucher) [58]
Parashar et al. 2011[41]	Not a Randomised Controlled Trial
Patterson et al. 2013[42]	No relevant outcomes reported
Piehler et al. 2014[43]	Intervention not qualifying – not providing permanent housing
Poremski et al. 2016[44]	No relevant outcomes reported
Rosenheck et al. 2003[45]	Intervention not qualifying – housing voucher can be denied in cases of continued use of controlled substances (section 8 housing voucher) [58]
Schutt et al. 1997[46]	Control not qualifying
Schutt et al. 2009[47]	Intervention not qualifying – housing voucher can be denied in cases of continued use of controlled substances (section 8 housing voucher) [58]
Shinn et al. 2016[48]	Intervention not qualifying – not providing permanent housing
Slesnick & Erdem 2013[49]	Intervention not qualifying – time-limited support
Somers, Patterson, et al. 2013[50]	No relevant outcomes reported

Somers, Rezansoff, et al. 2013[51]	No relevant outcomes reported
Sosin et al. 1995[52]	Intervention not qualifying – housing contingent on client accessing substance use services
Stahler et al. 1995[53]	Intervention not qualifying – client must 'make a complete commitment to treatment and sobriety' for 'residential treatment'
Stefancic & Tsemberis 2007[54]	No relevant outcomes reported
Tsemberis et al. 2003[55]	Intervention not qualifying
Veldhuizen et al. 2015[56]	No relevant outcomes reported
Westermeyer & Lee 2013[57]	Not a Randomised Controlled Trial
Wood et al. 1998[58]	Intervention not qualifying – housing voucher can be denied in cases of continued use of controlled substances (section 8 housing voucher) [59]

References

1. Adair CE, Kopp B, Distasio J, et al. Housing Quality in a Randomized Controlled Trial of Housing First for Homeless Individuals with Mental Illness: Correlates and Associations with Outcomes. *J Urban Health* 2016;93:682-97 doi:10.1007/s11524-016-0062-9.
2. Aubry T, Nelson G, Tsemberis S. Housing first for people with severe mental illness who are homeless: A review of the research and findings from the At Home-Chez soi demonstration project. *The Canadian Journal of Psychiatry / La Revue canadienne de psychiatrie* 2015;60(11):467-74.
3. Burnam MA, Morton SC, McGlynn EA, et al. An experimental evaluation of residential and nonresidential treatment for dually diagnosed homeless adults. *J Addict Dis* 1995;14(4):111-34.
4. Caplan B, Schutt RK, Turner WM, et al. Change in neurocognition by housing type and substance abuse among formerly homeless seriously mentally ill persons. *Schizophr Res* 2006;83(1):77-86 doi:10.1016/j.schres.2005.11.013.
5. Cheng A-L, Lin H, Kasprow W, et al. Impact of Supported Housing on Clinical Outcomes: Analysis of a Randomized Trial Using Multiple Imputation Technique. *J Nerv Ment Dis* 2007;195(1):83-88 doi:10.1097/01.nmd.0000252313.49043.f2.
6. Conrad KJ, Hultman CI, Pope AR, et al. Case managed residential care for homeless addicted veterans. Results of a true experiment. *Med Care* 1998;36(1):40-53.
7. Dickey B, Gonzalez O, Latimer E, et al. Use of mental health services by formerly homeless adults residing in group and independent housing. *Psychiatr Serv* 1996;47(2):152-8.
8. Dickey B, Latimer E, Powers K, et al. Housing costs for adults who are mentally ill and formerly homeless. *J Ment Health Adm* 1997;24(3):291-305 doi:10.1007/BF02832663.
9. Edens EL, Mares AS, Tsai J, et al. Does Active Substance Use at Housing Entry Impair Outcomes in Supported Housing for Chronically Homeless Persons? *Psychiatr Serv* 2011;62(2):171-78 doi:10.1176/appi.ps.62.2.171.
10. Fletcher TD, Cunningham JL, Calsyn RJ, et al. Evaluation of treatment programs for dual disorder individuals: Modeling longitudinal and mediation effects. *Administration and Policy in Mental Health and Mental Health Services Research* 2008;35(4):319-36 doi:10.1007/s10488-008-0170-2.
11. Forchuk C, MacClure SK, Van Beers M, et al. Developing and testing an intervention to prevent homelessness among individuals discharged from psychiatric wards to shelters and 'no fixed

address.'. *Journal of Psychiatric and Mental Health Nursing* 2008;15(7):569-75 doi:10.1111/j.1365-2850.2008.01266.x.
12. Fowler PJ, Chavira D. Family Unification Program: Housing Services for Homeless Child Welfare-Involved Families. *Housing Policy Debate* 2014;24(4):802-14 doi:10.1080/10511482.2014.881902.
13. Fowler PJ, Schoeny M. The Family Unification Program: A Randomized-Controlled Trial of Housing Stability. *Child Welfare* 2015;94(1):167-87.
14. Geller L. Putting housing first. *Can Nurse* 2014;110(5):22-7.
15. Gewirtz AH, DeGarmo DS, Lee S, et al. Two-year outcomes of the Early Risers prevention trial with formerly homeless families residing in supportive housing. *J Fam Psychol* 2015;29(2):242-52 doi:10.1037/fam0000066.
16. Goldfinger SM, Schutt RK, Tolomiczenko GS, et al. Housing placement and subsequent days homeless among formerly homeless adults with mental illness. *Psychiatr Serv* 1999;50(5):674-79 doi:10.1176/ps.50.5.674.
17. Greenwood RM, Schaefer-McDaniel NJ, Winkel G, et al. Decreasing Psychiatric Symptoms by Increasing Choice in Services for Adults with Histories of Homelessness. *Am J Community Psychol* 2005;36(3-4):223-38 doi:10.1007/s10464-005-8617-z.
18. Gulcur L, Tsemberis S, Stefancic A, et al. Community integration of adults with psychiatric disabilities and histories of homelessness. *Community Mental Health Journal* 2007;43(3):211-28 doi:10.1007/s10597-006-9073-4.
19. Guo X, Slesnick N, Feng X. Housing and support services with homeless mothers: Benefits to the mother and her children. *Community Ment Health J* 2016;52(1):73-83 doi:10.1007/s10597-015-9830-3.
20. Hurlburt MS, Hough RL, Wood PA. Effects of substance abuse on housing stability of homeless mentally ill persons in supported housing. *Psychiatr Serv* 1996;47(7):731-36 doi:10.1176/ps.47.7.731.
21. Kertesz SG, Mullins AN, Schumacher JE, et al. Long-term housing and work outcomes among treated cocaine-dependent homeless persons. *The Journal of Behavioral Health Services & Research* 2007;34(1):17-33 doi:10.1007/s11414-006-9041-3.
22. Korr WS, Joseph A. Housing the homeless mentally ill: Findings from Chicago. *Journal of Social Service Research* 1995;21(1):53-68 doi:10.1300/J079v21n01_04.
23. Kozloff N. Housing first "junior": Testing a complex psychosocial intervention designed for homeless adults with mental illness in homeless youth. *Journal of the American Academy of Child and Adolescent Psychiatry* 2016;55 (10 Supplement 1):S74.
24. Kozloff N, Stergiopoulos V, Cheung A, et al. Housing first for homeless youth with mental illness: Analysis from a randomized controlled trial. *Journal of the American Academy of Child and Adolescent Psychiatry* 2016;55 (10 Supplement 1):S149.
25. Krabbenborg MAM, Boersma SN, Beijersbergen MD, et al. Fidelity of a strengths-based intervention used by Dutch shelters for homeless young adults. *Psychiatr Serv* 2015;66(5):470-76 doi:10.1176/appi.ps.201300425.
26. Lapham SC, Hall M, McMurray-Avila M, et al. Albuquerque's community-based housing and support services demonstration program for homeless alcohol abusers. *Alcoholism Treatment Quarterly* 1993;10(3-4):139-54 doi:10.1300/J020V10N03_12.
27. Lapham SC, Hall M, Skipper BJ. Homelessness and substance use among alcohol abusers following participation in project H&ART. *J Addict Dis* 1995;14(4):41-55.
28. Levitt AJ, Mitchell K, Pareti L, et al. Randomized Trial of Intensive Housing Placement and Community Transition Services for Episodic and Recidivist Homeless Families. *Am J Public Health* 2013;103(Suppl. 2):S348-S54 doi:10.2105/ajph.2013.301521.
29. Malte CA, Cox K, Saxon AJ. Providing intensive addiction/housing case management to homeless veterans enrolled in addictions treatment: A randomized controlled trial. *Psychol Addict Behav* 2017;31(3):231-41 doi:10.1037/adb0000273.

30. Mares AS, Rosenheck RA. A Comparison of Treatment Outcomes Among Chronically Homelessness Adults Receiving Comprehensive Housing and Health Care Services Versus Usual Local Care. *Administration and Policy in Mental Health and Mental Health Services Research* 2011;38(6):459-75 doi:10.1007/s10488-011-0333-4.
31. McHugo GJ, Bebout RR, Harris M, et al. A Randomized controlled trial of integrated versus parallel housing services for homeless adults with severe mental illness. *Schizophr Bull* 2004;30(4):969-82.
32. Milby JB, Schumacher JE, Wallace D, et al. To house or not to house: The effects of providing housing to homeless substance abusers in treatment. *Am J Public Health* 2005;95(7):1259-65 doi:10.2105/ajph.2004.039743.
33. Morse GA, Calsyn RJ, Allen G, et al. Helping homeless mentally ill people: what variables mediate and moderate program effects? *Am J Community Psychol* 1994;22(5):661-83.
34. Morse GA, Calsyn RJ, Klinkenberg WD, et al. Treating homeless clients with severe mental illness and substance use disorders: Costs and outcomes. *Community Mental Health Journal* 2006;42(4):377-404 doi:10.1007/s10597-006-9050-y.
35. Morse GA, Calsyn RJ, Klinkenberg WD, et al. Integrated treatment for homeless clients with dual disorders: A quasi-experimental evaluation. *Journal of Dual Diagnosis* 2008;4(3):219-37.
36. Nelson G, Patterson M, Kirst M, et al. Life changes among homeless persons with mental illness: a longitudinal study of Housing First and usual treatment. *Psychiatr Serv* 2015;66(6):592-97 doi:10.1176/appi.ps.201400201.
37. O'Connell MJ, Kasprow W, Rosenheck RA. Rates and risk factors for homelessness after successful housing in a sample of formerly homeless veterans. *Psychiatr Serv* 2008;59(3):268-75.
38. O'Connell MJ, Kasprow WJ, Rosenheck RA. Differential impact of supported housing on selected subgroups of homeless veterans with substance abuse histories. *Psychiatric Services* 2012;63(12):1195-205 doi:10.1176/appi.ps.201000229.
39. O'Connell MJ, Kasprow WJ, Rosenheck RA. Impact of supported housing on social relationships among homeless veterans. *Psychiatr Serv* 2017;68(2):203-06.
40. Palepu A, Patterson M, Moniruzzaman A, et al. Housing first among homeless persons with concurrent disorders among participants of the Vancouver at home study. *J Gen Intern Med* 2013;28:S91.
41. Parashar S, Palmer AK, O'Brien N, et al. Sticking to It: The Effect of Maximally Assisted Therapy on Antiretroviral Treatment Adherence Among Individuals Living with HIV Who are Unstably Housed. *AIDS Behav* 2011;15(8):1612-22 doi:10.1007/s10461-011-0026-8.
42. Patterson ML, Rezansoff S, Currie L, et al. Trajectories of recovery among homeless adults with mental illness who participated in a randomised controlled trial of Housing First: A longitudinal, narrative analysis. *BMJ Open* 2013;3 (9) (no pagination)(e003442).
43. Piehler TF, Bloomquist ML, August GJ, et al. Executive functioning as a mediator of conduct problems prevention in children of homeless families residing in temporary supportive housing: A parallel process latent growth modeling approach. *J Abnorm Child Psychol* 2014;42(5):681-92 doi:10.1007/s10802-013-9816-y.
44. Poremski D, Stergiopoulos V, Braithwaite E, et al. Effects of Housing First on Employment and Income of Homeless Individuals: Results of a Randomized Trial. *Psychiatric services (Washington, DC)* 2016;67(6):603-9 doi:10.1176/appi.ps.201500002 [published Online First: 2016/02/16].
45. Rosenheck R, Kasprow W, Frisman L, et al. Cost-effectiveness of supported housing for homeless persons with mental illness. *Arch Gen Psychiatry* 2003;60(9):940-51 doi:10.1001/archpsyc.60.9.940.
46. Schutt RK, Goldfinger SM, Penk WE. Satisfaction with residence and with life: When homeless mentally ill persons are housed. *Eval Program Plann* 1997;20(2):185-94 doi:10.1016/S0149-7189(96)00049-3.

47. Schutt RK, Hough RL, Goldfinger SM, et al. Lessening Homelessness Among Persons with Mental Illness: A Comparison of Five Randomized Treatment Trials. *Asian journal of psychiatry* 2009;2(3):100-02 doi:10.1016/j.ajp.2009.07.003 [published Online First: 2010/02/18].
48. Shinn M, Brown SR, Gubits D. Can housing and service interventions reduce family separations for families who experience homelessness? *Am J Community Psychol* 2016 doi:10.1002/ajcp.12111.
49. Slesnick N, Erdem G. Efficacy of ecologically-based treatment with substance-abusing homeless mothers: Substance use and housing outcomes. *J Subst Abuse Treat* 2013;45(5):416-25 doi:10.1016/j.jsat.2013.05.008.
50. Somers JM, Patterson ML, Moniruzzaman A, et al. Vancouver At Home: pragmatic randomized trials investigating Housing First for homeless and mentally ill adults. *Trials [Electronic Resource]* 2013;14:365.
51. Somers JM, Rezansoff SN, Moniruzzaman A, et al. Housing First Reduces Re-offending among Formerly Homeless Adults with Mental Disorders: Results of a Randomized Controlled Trial. *PLoS One* 2013;8(9):e72946 doi:10.1371/journal.pone.0072946.
52. Sosin MR, Bruni M, Reidy M. Paths and impacts in the progressive independence model: a homelessness and substance abuse intervention in Chicago. *J Addict Dis* 1995;14(4):1-20.
53. Stahler GJ, Shipley TF, Jr., Bartelt D, et al. Evaluating alternative treatments for homeless substance-abusing men: outcomes and predictors of success. *J Addict Dis* 1995;14(4):151-67.
54. Stefancic A, Tsemberis S. Housing first for long-term shelter dwellers with psychiatric disabilities in a suburban county: A four-year study of housing access and retention. *The Journal of Primary Prevention* 2007;28(3-4):265-79 doi:10.1007/s10935-007-0093-9.
55. Tsemberis SJ, Moran L, Shinn M, et al. Consumer preference programs for individuals who are homeless and have psychiatric disabilities: A drop-in center and a supported housing program. *Am J Community Psychol* 2003;32(3-4):305-17 doi:10.1023/B:AJCP.0000004750.66957.bf.
56. Veldhuizen S, Adair CE, Methot C, et al. Patterns and predictors of attrition in a trial of a housing intervention for homeless people with mental illness. *Soc Psychiatry Psychiatr Epidemiol* 2015;50(2):195-202 doi:10.1007/s00127-014-0909-x.
57. Westermeyer J, Lee K. Residential Placement for Veterans With Addiction American Society of Addiction Medicine Criteria vs. a Veterans Homeless Program. *J Nerv Ment Dis* 2013;201(7):567-71 doi:10.1097/NMD.0b013e3182982d1a.
58. Wood PA, Hurlburt MS, Hough RL, et al. Longitudinal assessment of family support among homeless mentally ill participants in a supported housing program. *J Community Psychol* 1998;26(4):327-44 doi:10.1002/(SICI)1520-6629(199807)26:4<327::AID-JCOP3>3.0.CO;2-Q.
59. U.S. Department of Housing and Urban Development. Chapter 5: Eligibility and Denial of Assistance. Housing Choice Voucher Program Guidebook, 2001.
