# Supplementary material for: Effects of Housing First approaches on health and well-being of adults who are homeless or at risk of homelessness: systematic review and meta-analysis of randomised controlled trials
Source: J Epidemiol Community Health. 2019 Feb 18;73(5):379–87. doi: 10.1136/jech-2018-210981 (PMC6581117; doi:10.1136/jech-2018-210981)
Supplement: Supplementary data [file jech-2018-210981supp004.pdf]

Supplementary File 4 – Risk of Bias table; Forest plots presenting standard effect sizes of secondary housing stability outcomes and subgroup data of At Home/Chez Soi study in intervention groups compared to control

Table 3 – Risk of Bias of the four included studies across the outcome domains.

Study	*Outcomes assessed*	*Bias arising from the randomisation process*	*Bias due to deviations from the intended interventions*	*Bias due to missing outcome data*	*Bias in measurement of the outcome*	*Bias in the selection of the reported result*	***Overall RoB judgement***
Pathways Housing First	Substance Use	Low	Some Concerns	Low	High	Low	High
	Mental Health	Low	Some Concerns	Low	High	Low	High
	Health Service Use	Low	Some Concerns	Low	High	Low	High
	Housing Stability	Low	High	High	High	Low	High
At Home/Chez Soi	Substance Use	Low	Some Concerns	High	High	Low	High
	Mental Health	Low	Some Concerns	High	High	Low	High
	Quality of Life	Low	Some Concerns	High	High	Low	High
	Health Service Use	Low	Some Concerns	High	High	Low	High
	Housing Stability	Low	High	High	High	Low	High
HOPWA	Mental Health	Low	Some Concerns	Low	High	Low	High
	Quality of Life	Low	Some Concerns	Low	High	Low	High
	Health Service Use	Low	Some Concerns	Low	High	Low	High
	Housing Stability	Low	High	High	High	Low	High
CHHP	Mental Health	Low	Some Concerns	High	High	Low	High
	Quality of Life	Low	Some Concerns	High	High	Low	High
	Health Service Use	Low	Some Concerns	High	High	Low	High
	Housing Stability	Low	High	High	High	Low	High

Forest plots of Housing Stability and subgroup comparisons

a) Likelihood (Risk Ratio) of achieving stable housing at 24 months

Study	Intervention Total	Control Total	Weight	RR IV, Random, 95% CI
At Home - High Needs	469	481	36.1%	1.80 [1.57, 2.07]
CHHP	201	204	27.7%	6.30 [4.09, 9.69]
HOPWA	315	315	36.1%	1.63 [1.42, 1.87]
**Total (95% CI)**	**985**	**1000**	**100.0%**	**2.46 [1.58, 3.84]**

Heterogeneity: $I^2 = 94\%$
Test for overall effect: $P < 0.0001$

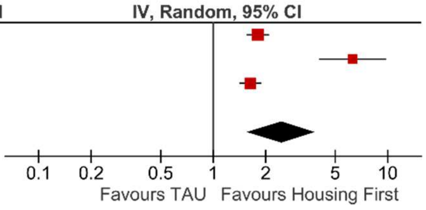

b) Standardised mean difference in time spent stably housed in 24 months

Study	Intervention Total	Control Total	Weight	SMD IV, Random, 95% CI
At Home - 18-49 age subgroup	905	773	36.2%	1.36 [1.25, 1.48]
At Home - 50+ age subgroup	253	217	32.9%	1.57 [1.34, 1.80]
Pathways Housing First	99	126	30.9%	0.74 [0.46, 1.03]
**Total (95% CI)**	**1257**	**1116**	**100.0%**	**1.24 [0.86, 1.62]**

Heterogeneity: $I^2 = 90\%$
Test for overall effect: $P < 0.00001$

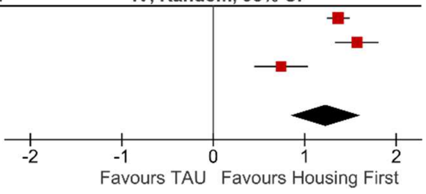

c) Subgroup analysis of standardised mean difference in improvement in generic quality of life score at 24 months

Subgroup	Intervention Total	Control Total	SMD IV, Random, 95% CI
**Stratified by age**			
At Home - 18-49 age subgroup	905	773	-0.04 [-0.15, 0.06]
At Home - 50+ age subgroup	253	217	0.01 [-0.19, 0.22]
**Stratified by high/moderate needs**			
At Home - High Needs	469	481	-0.17 [-0.36, 0.03]
At Home - Moderate Needs	689	509	0.00 [-0.12, 0.13]
**Stratified by CHF/SHF**			
At Home - CHF	107	100	0.05 [-0.23, 0.32]
At Home - SHF	90	100	-0.11 [-0.40, 0.18]

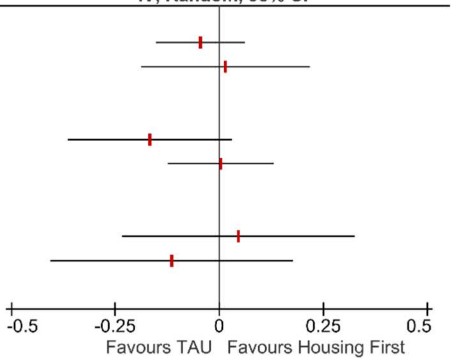

d) Subgroup analysis of standardised mean difference in changes in condition-specific quality of life score at 24 months

Subgroup	Intervention Total	Control Total	SMD IV, Random, 95% CI
**Stratified by age**			
At Home - 18-49 age subgroup	905	773	0.06 [-0.05, 0.16]
At Home - 50+ age subgroup	253	217	0.34 [0.13, 0.54]
**Stratified by high/moderate needs**			
At Home - High Needs	469	481	0.03 [-0.17, 0.22]
At Home - Moderate Needs	689	509	0.20 [0.07, 0.32]
**Stratified by CHF/SHF**			
At Home - CHF	107	100	0.21 [-0.07, 0.49]
At Home - SHF	90	100	0.16 [-0.13, 0.45]

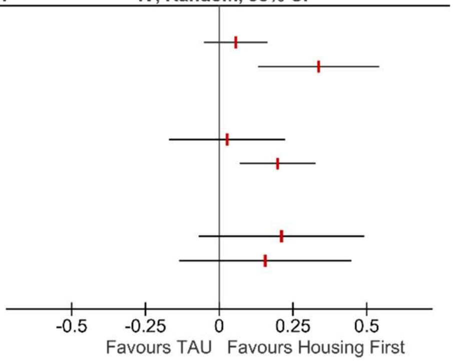

e) Subgroup analysis of standardised mean difference in changes in mental health symptom severity score at 24 months

Subgroup	Intervention Total	Control Total	SMD IV, Random, 95% CI
**Stratified by age**			
At Home - 18-49 age subgroup	673	617	0.17 [0.06, 0.28]
At Home - 50+ age subgroup	216	180	-0.10 [-0.30, 0.09]
**Stratified by high/moderate needs**			
At Home - High Needs	469	481	0.16 [-0.03, 0.36]
At Home - Moderate Needs	689	509	0.05 [-0.08, 0.18]
**Stratified by CHF/SHF**			
At Home - CHF	107	100	0.11 [-0.16, 0.39]
At Home - SHF	90	100	0.26 [-0.03, 0.55]

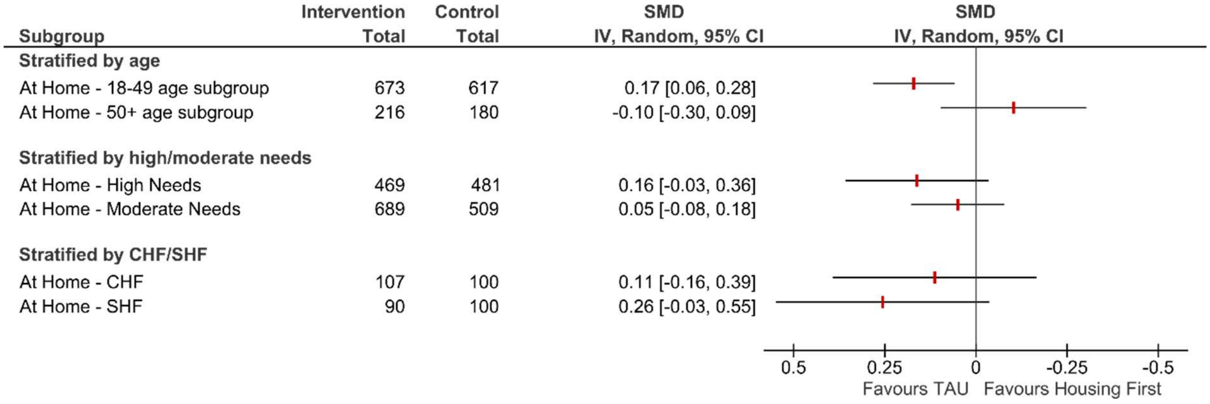

f) Subgroup analysis of Ratio of Rate Ratios of substance use problems at 24-month followup compared to baseline

Subgroup	Intervention Total	Control Total	RRR IV, Random, 95% CI
**Stratified by age**			
At Home - 18-49 age subgroup	905	773	1.07 [0.93, 1.23]
At Home - 50+ age subgroup	253	217	0.79 [0.56, 1.11]
**Stratified by high/moderate needs**			
At Home - High Needs	469	481	1.13 [0.98, 1.32]
At Home - Moderate Needs	689	509	0.94 [0.79, 1.12]
**Stratified by CHF/SHF**			
At Home - CHF	107	100	1.28 [0.94, 1.76]
At Home - SHF	90	100	1.29 [0.92, 1.81]

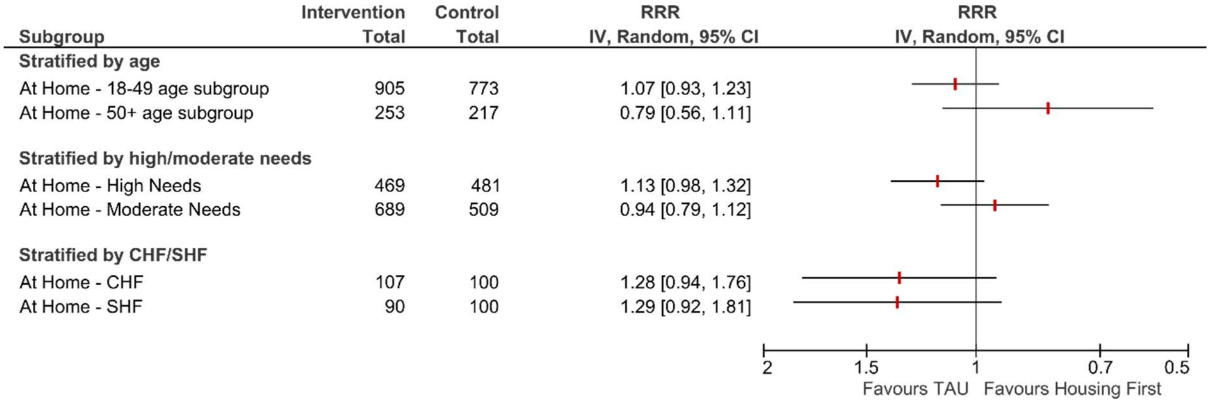

RR: Risk Ratio; SMD: Standardised mean difference; RRR: Ratio of Ratio Ratios; CHHP: Chicago Housing for Health Partnership; HOPWA: Housing Opportunities for Persons With AIDS; TAU: Treatment as usual; CHF: Congregate Housing First (HF clients housed in separate accomodation within same building); SHF: Scattered-site Housing First (no more than 30% of appartments in a building allocated to HF clients)
