# Supplementary material for: Effects of Housing First approaches on health and well-being of adults who are homeless or at risk of homelessness: systematic review and meta-analysis of randomised controlled trials
Source: J Epidemiol Community Health. 2019 Feb 18;73(5):379–87. doi: 10.1136/jech-2018-210981 (PMC6581117; doi:10.1136/jech-2018-210981)
Supplement: Supplementary data [file jech-2018-210981supp005.pdf]

Supplementary File 5 – Other outcomes

Paper	Study	Outcome	Measure reported	Result as reported (95%CI or P)
Aubry et al. 2016[1]	At Home (HN subgroup)	Community Functioning (MCAS)	Difference in mean changes from baseline	SMD=0.12 (-0.04 to 0.30), favouring Housing First
Aubry et al. 2016[1]	At Home (HN subgroup)	Physical Community Integration (CIS)	Difference in mean changes from baseline	No statistically significant changes from baseline in both groups
Aubry et al. 2016[1]	At Home (HN subgroup)	Psychological Integration (CIS)	Difference in mean changes from baseline	Both groups reporting significant improvements – not statistically significant between groups
Chung et al. 2017[2]	At Home (≥50 subgroup)	Community Functioning (MCAS)	Difference (D) in mean changes from baseline	D=0.70 (-1.19 to 2.59), favouring Housing First
Chung et al. 2017[2]	At Home (18-49 subgroup)	Community Functioning (MCAS)	Difference in mean changes from baseline	D=0.40 (-0.60 to 1.40), favouring Housing First
Chung et al. 2017[2]	At Home (≥50 subgroup)	Psychological Community Integration (CIS)	Difference in mean changes from baseline	D=0.16 (-0.73 to 1.05), favouring Housing First
Chung et al. 2017[2]	At Home (18-49 subgroup)	Psychological Community Integration (CIS)	Difference in mean changes from baseline	D=0.24 (-0.23 to 0.72), favouring Housing First
Chung et al. 2017[2]	At Home (≥50 subgroup)	Recovery (RAS)	Difference in mean changes from baseline	D=2.21 (-0.74 to 5.16), favouring Housing First
Chung et al. 2017[2]	At Home (18-49 subgroup)	Recovery (RAS)	Difference in mean changes from baseline	D=-0.57 (-2.11 to 0.97), favouring TAU
Kozloff 2016[3]	At Home (18-24 subgroup)	Community Functioning (MCAS)	Difference in mean changes from baseline	D=0.25 (-2.79 to 3.28), favouring Housing First
Kozloff 2016[3]	At Home (18-24 subgroup)	Psychological Community Integration (CIS)	Difference in mean changes from baseline	D=0.49 (-0.99 to 1.98), favouring Housing First
Kozloff 2016[3]	At Home (18-24 subgroup)	Recovery (RAS)	Difference in mean changes from baseline	D=1.80 (-3.33 to 6.93), favouring Housing First
Parpouchi et al. 2016[4]	At Home	Unprotected sex	Rates	adjusted OR=1.00 (0.71 to 1.43)

Rezansoff et al. 2016[5]	At Home (CHF subgroup, diagnosed schizophrenia)	Adherence to anti-psychotic medication	Difference in ratio of adherence to anti-psychotic medication	D=0.06 (-0.10 to 0.21), favouring Housing First
Rezansoff et al. 2016[5]	At Home (SHF subgroup, diagnosed schizophrenia)	Adherence to anti-psychotic medication	Difference in ratio of adherence to anti-psychotic medication	D=0.24 (0.10 to 0.37), favouring Housing First
Somers et al. 2017[6]	At Home (Vancouver, Overall)	Community Functioning (MCAS)	Difference in mean changes from baseline (P value only)	P<0.001, favouring Housing First
Somers et al. 2017[6]	At Home (CHF subgroup)	Community Functioning (MCAS)	Difference in mean changes from baseline	D=5.81 (2.69 to 8.93), favouring Housing First
Somers et al. 2017[6]	At Home (SHF subgroup)	Community Functioning (MCAS)	Difference in mean changes from baseline	D=1.66 (-1.59 to 4.92), favouring Housing First
Somers et al. 2017[6]	At Home (Vancouver, Overall)	Physical Community Integration (CIS)	Difference in mean changes from baseline (P value only)	P=0.002
Somers et al. 2017[6]	At Home (CHF subgroup)	Physical Community Integration (CIS)	Difference in mean changes from baseline	D=0.47 (-0.14 to 1.09), favouring Housing First
Somers et al. 2017[6]	At Home (SHF subgroup)	Physical Community Integration (CIS)	Difference in mean changes from baseline	D=-0.53 (-1.16 to 0.11), favouring TAU
Somers et al. 2017[6]	At Home (Vancouver, Overall)	Psychological Community Integration (CIS)	Difference in mean changes from baseline (P value only)	P<0.001, favouring Housing First
Somers et al. 2017[6]	At Home (CHF subgroup)	Psychological Community Integration (CIS)	Difference in mean changes from baseline	D=2.53 (1.05 to 4.01), favouring Housing First
Somers et al. 2017[6]	At Home (SHF subgroup)	Psychological Community Integration (CIS)	Difference in mean changes from baseline	D=-0.34 (-1.88 to 1.20), favouring TAU
Somers et al. 2017[6]	At Home (Vancouver, Overall)	Recovery (RAS)	Difference in mean changes from baseline (P value only)	P=0.0025, favouring Housing First
Somers et al. 2017[6]	At Home (CHF subgroup)	Recovery (RAS)	Difference in mean changes from baseline	D=5.58 (1.65 to 9.50), favouring Housing First

Somers et al. 2017[6]	At Home (SHF subgroup)	Recovery (RAS)	Difference in mean changes from baseline	Difference in change of score=0.05 (-3.63 to 3.74), favouring Housing First
Stergiopoulos et al. 2015[7]	At Home (MN subgroup)	Community Functioning (MCAS)	Difference in mean changes from baseline	D=1.06 (0 to 2.13), favouring Housing First
Stergiopoulos et al. 2015[7]	At Home (MN subgroup)	Physical Community Integration (CIS)	Ratio of Rate Ratios	RRR=1.02 (0.92 to 1.14), favouring Housing First
Stergiopoulos et al. 2015[7]	At Home (MN subgroup)	Psychological Community Integration (CIS)	Difference in mean changes from baseline	D=0.31 (-0.25 to 0.86), favouring Housing First
Stergiopoulos et al. 2015[7]	At Home (MN subgroup)	Recovery (RAS)	Difference in mean changes from baseline	D=0.09 (-1.53 to 1.71), favouring Housing First
Woodhall-Melnik et al. 2015[8]	At Home (Toronto subgroup)	Body Mass Index (BMI)	Variations in changes from baseline	MN group B=0.00063 (P=0.99)<br>HN group B=0.91 (P=0.34)
Woodhall-Melnik et al. 2015[8]	At Home (Toronto subgroup)	Waist circumference	Variations in changes from baseline	MN group β=1.01 (P=0.52)<br>HN group β=2.10 (P=0.64)
Buchanan et al. 2009[9]	CHHP (HIV+ subgroup)	Survival with intact immunity	Relative Risk	RR=1.63 (1.01 to 2.61), favouring Housing First
Buchanan et al. 2009[9]	CHHP (HIV+ subgroup)	Undetectable viral load	Relative Risk	RR=1.93 (0.97 to 3.84), favouring Housing First
Wolitski et al. 2010[10]	HOPWA	CD4 below 200	Variations in changes from baseline	F=0.11 (P=0.9522)
Wolitski et al. 2010[10]	HOPWA	Detectable viral load	Variations in changes from baseline	F=1.03 (P=0.3770)
Wolitski et al. 2010[10]	HOPWA	Health risk behaviour (past three months)	Variations in changes from baseline	F=2.26 (P=0.0801)

SMD: Standardised mean difference; OR: Odds ratio; RR: Relative risk; RRR: Ratio of rate ratios; MCAS: Multnomah Community Ability Scale; CIS: Community Integration Scale; RAS: Recovery Assessment Scale; MN: Moderate needs subgroup; HN: High needs subgroup; CHF: Congregate Housing First subgroup; SHF: Scattered-site Housing First subgroup; CHHP: Chicago Housing for Health Partnership; HOPWA: Housing Opportunities for Persons With AIDS

References

1. Aubry T, Goering P, Veldhuizen S, et al. A multiple-city RCT of housing first with assertive community treatment for homeless Canadians with serious mental illness. *Psychiatr Serv* 2016;67:275-81 doi:10.1176/appi.ps.201400587 [published Online First: 1 December 2015].
2. Chung TE, Gozdzik A, Palma Lazgare LI, et al. Housing first for older homeless adults with mental illness: A subgroup analysis of the at home/chez soi randomized controlled trial. *Int J Geriatr Psychiatry* 2017 doi:10.1002/gps.4682 [published Online First: 16 February 2017].
3. Kozloff N, Adair CE, Lazgare LIP, et al. "Housing First" for Homeless Youth With Mental Illness. *Pediatrics* 2016;138:e20161514 doi:10.1542/peds.2016-1514 [published Online First: 30 September 2016].

4. Parpouchi M, Moniruzzaman A, McCandless L, et al. Housing First and Unprotected Sex: A Structural Intervention. *J Health Care Poor Underserved* 2016;27(3):1278-302 doi:10.1353/hpu.2016.0113 [published Online First: 2016/08/16].
5. Rezansoff SN, Moniruzzaman A, Fazel S, et al. Housing First Improves Adherence to Antipsychotic Medication Among Formerly Homeless Adults With Schizophrenia: Results of a Randomized Controlled Trial. *Schizophr Bull* 2016 doi:10.1093/schbul/sbw136 [published Online First: 2016/09/25].
6. Somers JM, Moniruzzaman A, Patterson M, et al. A Randomized Trial Examining Housing First in Congregate and Scattered Site Formats. *PLoS One* 2017;12(1):e0168745 doi:10.1371/journal.pone.0168745 [published Online First: 11 Jan 2017].
7. Stergiopoulos V, Hwang SW, Gozdzik A, et al. Effect of Scattered-Site Housing Using Rent Supplements and Intensive Case Management on Housing Stability Among Homeless Adults With Mental Illness A Randomized Trial. *Jama-Journal of the American Medical Association* 2015;313(9):905-15 doi:10.1001/jama.2015.1163.
8. Woodhall-Melnik J, Misir V, Kaufman-Shriqui V, et al. The Impact of a 24 Month Housing First Intervention on Participants' Body Mass Index and Waist Circumference: Results from the At Home/Chez Soi Toronto Site Randomized Controlled Trial. *PLoS One* 2015;10(9):e0137069 doi:10.1371/journal.pone.0137069.
9. Buchanan D, Kee R, Sadowski LS, et al. The health impact of supportive housing for HIV-positive homeless patients: a randomized controlled trial. *Am J Public Health* 2009;99 Suppl 3:S675-80 doi:10.2105/AJPH.2008.137810 [published Online First: 16 April 2009].
10. Wolitski RJ, Kidder DP, Pals SL, et al. Randomized trial of the effects of housing assistance on the health and risk behaviors of homeless and unstably housed people living with HIV. *AIDS Behav* 2010;14(3):493-503 doi:10.1007/s10461-009-9643-x [published Online First: 1 December 2009].
